# Perampanel Improves Cortical Myoclonus and Disability in Progressive Myoclonic Epilepsies: A Case Series and a Systematic Review of the Literature

**DOI:** 10.3389/fneur.2021.630366

**Published:** 2021-03-24

**Authors:** Giovanni Assenza, Cristofaro Nocerino, Mario Tombini, Giancarlo Di Gennaro, Alfredo D'Aniello, Alberto Verrotti, Alfonso Marrelli, Lorenzo Ricci, Jacopo Lanzone, Vincenzo Di Lazzaro, Leonilda Bilo, Antonietta Coppola

**Affiliations:** ^1^Unit of Neurology, Neurophysiology and Neurobiology, Department of Medicine, Università Campus Bio-Medico di Roma, Rome, Italy; ^2^Department of Neurosciences, Reproductive Sciences and Odontostomatology, University Federico II of Naples, Naples, Italy; ^3^Epilepsy Surgery Center, IRCCS NEUROMED, Pozzilli, Italy; ^4^Department of Pediatrics, University of Perugia, Perugia, Italy; ^5^Clinical Neurophysiology Unit -Epilepsy Center, San Salvatore Hospital, L'Aquila, Italy

**Keywords:** progressive myoclonic epilepsy, perampanel, myoclonus, disability, systematic (literature) review

## Abstract

**Introduction:** Progressive myoclonic epilepsies (PMEs) are a heterogenous group of genetic diseases presenting with epilepsy, cognitive impairment, and severe action myoclonus, which can severely affect daily life activities and independent walking ability. Perampanel is a recent commercially available antiseizure medication with high efficacy against generalized seizures. Some reports supported the role of perampanel in ameliorating action myoclonus in PMEs. Here, we aimed to describe a case series and provide a systematic literature review on perampanel effects on PMEs.

**Methods:** We report the perampanel effectiveness on myoclonus, daily life activities, and seizures on an original Italian multicenter case series of 11 individuals with PMEs. Then, using the Preferred Reporting Items for Systematic Reviews and Meta-analyses (PRISMA) guidelines, we performed a systematic review on perampanel effect on myoclonus and disability in PMEs. We searched PubMed, Scopus, and Google Scholar articles on perampanel and PMEs up to June 2020. No prospective trials were found. We reviewed 11 case series manuscripts reporting 104 cases of different PMEs.

**Results:** Here, we are reporting the effectiveness of perampanel in five individuals affected by Unverricht–Lundborg disease, three by Lafora disease, two by sialidosis, and one by an undetermined PME. Nine out of 11 individuals improved their disability related to the action myoclonus (two with Lafora disease did not). Among the 104 persons with PMEs collected by the systematic review, we found that more than half of the patients receiving perampanel exhibited an amelioration of action myoclonus and, consequently, of their independence in daily life activities. The Unverricht–Lundborg disease seemed to show the best clinical response to perampanel, in comparison with the other more severe PMEs. A significant seizure reduction was achieved by almost all persons with active epilepsy. Only 11% of PME patients dropped out due to inefficacy.

**Conclusions:** Perampanel demonstrated a beneficial effect with regard to action myoclonus, disability, and seizures and was well-tolerated in people with PMEs, independently from their genetic diagnosis. Given the limited scientific evidence, broader prospective trials should be encouraged.

## Introduction

Progressive myoclonic epilepsies (PMEs) are a group of genetic disorders characterized by epilepsy, cognitive impairment, and severe cortical myoclonus, which heterogeneously combines their severity across different forms. PMEs range from milder diseases as the Unverricht–Lundborg disease (ULD), which present in infancy with generalized seizures followed by myoclonus, ataxia, cognitive impairment, and a consequent loss of independence in the second or third decades of life despite a standard life span, and more severe PMEs such as Lafora disease, sialidoses, and other rare disorders, which present with severe disability and reduction in life expectancy (1).

Myoclonus is often fragmentary and multifocal, involving the musculature of the face and distal limbs, but generalized massive proximal myoclonic jerks may frequently occur causing falls. Myoclonus may be spontaneous and reflexed to multisensorial stimuli. However, action myoclonus is the most frequent, and its drug resistance is the main cause of disability in daily life and, thus, in quality of life of PME patients (2).

At present, the best treatment of PMEs remains symptomatic, with antiseizure medications (ASMs) that are efficacious in controlling the myoclonus and the seizures (3). Valproic acid is one of the best options because it is often effective in suppressing seizures, photic sensitivity, and myoclonus. Barbiturates (phenobarbital and primidone) are effective but are burdened with cognitive impairment. Levetiracetam, piracetam, topiramate, zonisamide, and benzodiazepines provide a good, often transient, antimyoclonic effects. Ethosuximide and felbamate can also be effective, while Na-channel blockers can worsen myoclonus (3).

Perampanel is an ASM with an innovative mechanism of action, as it selectively inhibits the methyl-4-isoxazolepropionic acid (AMPA) receptors. Perampanel is efficacious in genetic generalized epilepsies (4), thus suggesting its role in inhibiting cortico-subcortical synchronization pathways favoring a diffuse increase in cortical excitability. These preclinical and clinical data suggested the use of perampanel in PME with very encouraging results, not only for seizure controls but also and particularly in mitigating cortical myoclonus and thus the disability of patients.

In the present paper, we report our experience and review all the available reported cases in the literature about the perampanel efficacy in controlling the myoclonus and improving the disability in activity of daily life in PME patients. Taken together, these results can encourage its use in PME.

## Case Descriptions

We reported a multicenter case series of 11 patients affected by different forms of PMEs. Clinical data are reported in Table 1. Briefly, we collected clinical data before and after the introduction of perampanel focusing on myoclonus, disability on activity of daily life (ADL), and seizures (Table 1).

**Table 1:** Demographic and clinical data.

**Patient *N*, medical center**	**Gender/Age**	**Pme type**	**Age at onset**	**Pme duration**	**Asms (mg/die)**	**Per dose (mg/die)**	**Gtcs^Pre^ (year)**	**Gtcs^post^ (year)**	**Disability^pre^ (smrs)**	**Disability^post^ (smrs)**	**F-up (months)**	**Benefits until F-UP**
1, UFIIN	M/40	ULD	10	30	VPA 1,500 TPM 150 CLZ 10 PIR 10,800	4	0	0	2	1	12	Yes
2, UFIIN	M/38	ULD	10	28	VPA 1,000 TPM1 50 CLZ 10 PIR 10,800	4	0	0	3	2	14	Yes
3, UFIIN	M/29	ULD	10	19	VPA 1,500 TPM 400 CLZ 6 CLB 20 PIR 10,800	8	0	0	4	2	14	Yes
4, UCBM	M/47	ULD	9	38	PB 100 VPA 500 LEV 2,000 ACT 125 PIR 12,000 CLZ 10	10	0	0	4	3	19	Yes
5, UCBM	M/46	ULD	13	33	VPA 1,000 ZNS 500 PIR 12,000 CLZ 20	6	0	0	4	2	16	Yes
6, UFIIN	F/26	Sialidosis type 1	13	13	LEV 1,500 ACT 250 CLN 3,000	4	0	0	5	4	3	Yes
7, UFIIN	F/18	Sialidosis type 1	12	6	LEV 1,000 ACT 250	4	0	0	4	3	6	Yes
8, UFIIN	M/34	Undetermined	8	26	VPA 1,500 CLN 1,800 LEV 1,500 mg	4	2	0	3	2	3	Yes
9, NMD	M/25	Lafora disease	10	15	VPA 2,000 LEV 3,000 CLN 4,000	10	1	0.3	5	3	16	No
10, NMD	M/19	Lafora disease	12	7	VPA 1,500 LEV 3,000 CLN 4,000 ZNS 400	8	1	1	3	3	6	No benefits
11, USA	M/16	Laforadisease	4	8	LEV 1,000 VPA 750 CLB 10	6	0.8	0.6	5	5	24	No benefits

*Medical centers: UFIIN, University Federico II Naples; UCBM, Università Campus Bio-Medico di Roma; NMD, Neuromed: Università degli Studi dell'Aquila*.

*M, male; F, female; ULD, Unverricht-Lundborg disease; ASM, anti-seizure medications; F-UP, follow-up; PRE, pre-Perampanel; POST, post-Perampanel; GTCS, Generalized tonic-clonic seizures; CLN, clonazepam; BVR, brivaracetam; LEV, levetiracetam; PIR, piracetam; VPA, valproate; ZNS, zonisamide. ACT, acetazolamide; CLB, clobazam; PER, perampanel; F-UP, follow-up; SMRS, Simplified Myoclonus Rating Scale (5)*.

We reported five individuals (five male, aged 29–47 years) affected by ULD. Three of them were siblings (patients 1–3). They had the classic mutation with dodecamer expansion in the cystatin B gene on chromosome 21q. These patients had an onset during infancy/early adolescence (9–13 years) and had a long history of disease at the moment of perampanel administration (19–38 years). At the moment of this case description, none of them suffered from generalized tonic–clonic seizures (GTCSs), but all presented action myoclonus. One patient was able to walk independently [simplified myoclonus ranking scale (SMRS), score = 2; patient 1, Table 1), while one of them presented a gait disturbance requiring support (SMRS = 3, patient 2), and three patients were wheelchair bound (SMRS = 4, patients 3–5). Perampanel was added to four ASMs or more at a dose of 4–10 mg/day. All patients demonstrated an improvement of the action myoclonus and referred a marked amelioration of autonomy in ADL, as they gained 1–2 points at the SMRS scoring. At the follow-up visits (12–19 months), patients preserved their clinical benefit.

Three young adult male patients with 7–15-year history of Lafora disease and treated with three to four ASMs were prescribed perampanel 6–10 mg/day. Perampanel did not change GTCS frequency in one patient, reduced GTCS frequency by 30% in one patient and by 66% in the other patient. This last patient also reported a clinical amelioration of action myoclonus and related disability (SMRS improved from 5 to 3 score), but it was only of a transient nature. Indeed, the beneficial effects progressively disappeared as demonstrated at the follow-up examination after 6 months of treatment.

**Figure 1:**
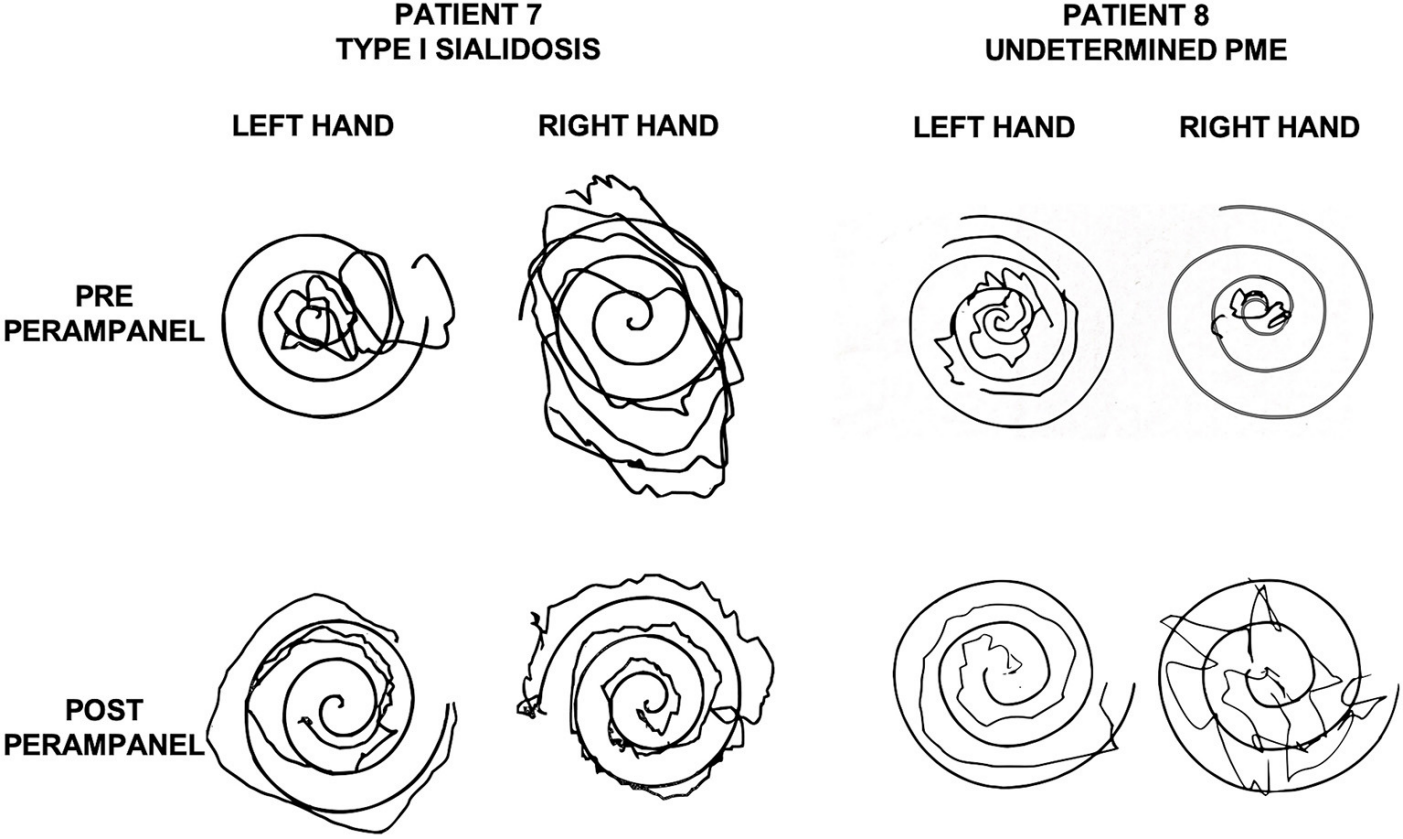
Archimedes' spirals executed by a patient with Sialidosis type 1 (patient 7, Table 1; left) and by a patient with undetermined PME (patient 8; Table 1; right) before and after perampanel. Please note the improvement after perampanel treatment. PME, progressive myoclonic epilepsy.

In two young adult female patients with type 1 sialidosis, perampanel 4 mg/day was added to their two ASMs. They did not complain any recent GTCS but were severely disabled because of the action myoclonus, which significantly improved after perampanel introduction. The myoclonus was evaluated by using the Unified Myoclonus Rating Scale (UMRS) before perampanel administration and at the 6-month follow-up. The first patient went from a total score of 144 before perampanel introduction to a total score of 100 at 6 months follow-up (see Supplementary Video 1). The second patient went from a total score of 102 to a total score of 50 at 6 months follow-up (see Figure 1).

In one young male patient with undetermined PME, perampanel 4 mg/day was added to his treatment regimen (valproic acid, 1,500 mg/day; clonazepam, 1,500 mg/day; levetiracetam, 1,500 mg/day). No GTCSs were recorded at 3 months follow-up. However, pre-perampanel treatment, GTCS frequency was pretty low, and thus, it was not possible to determine the antiseizure effect of perampanel. This patient presented with a moderate myoclonus after 3 months of perampanel 4 mg/day treatment and reported a significant benefit with regard to the action myoclonus (UMRS total score pre-perampanel was 84; at 3 months follow-up, it was 46).

## Literature Review

### Literature Search Strategy and Study Selection Process

**Figure 2:**
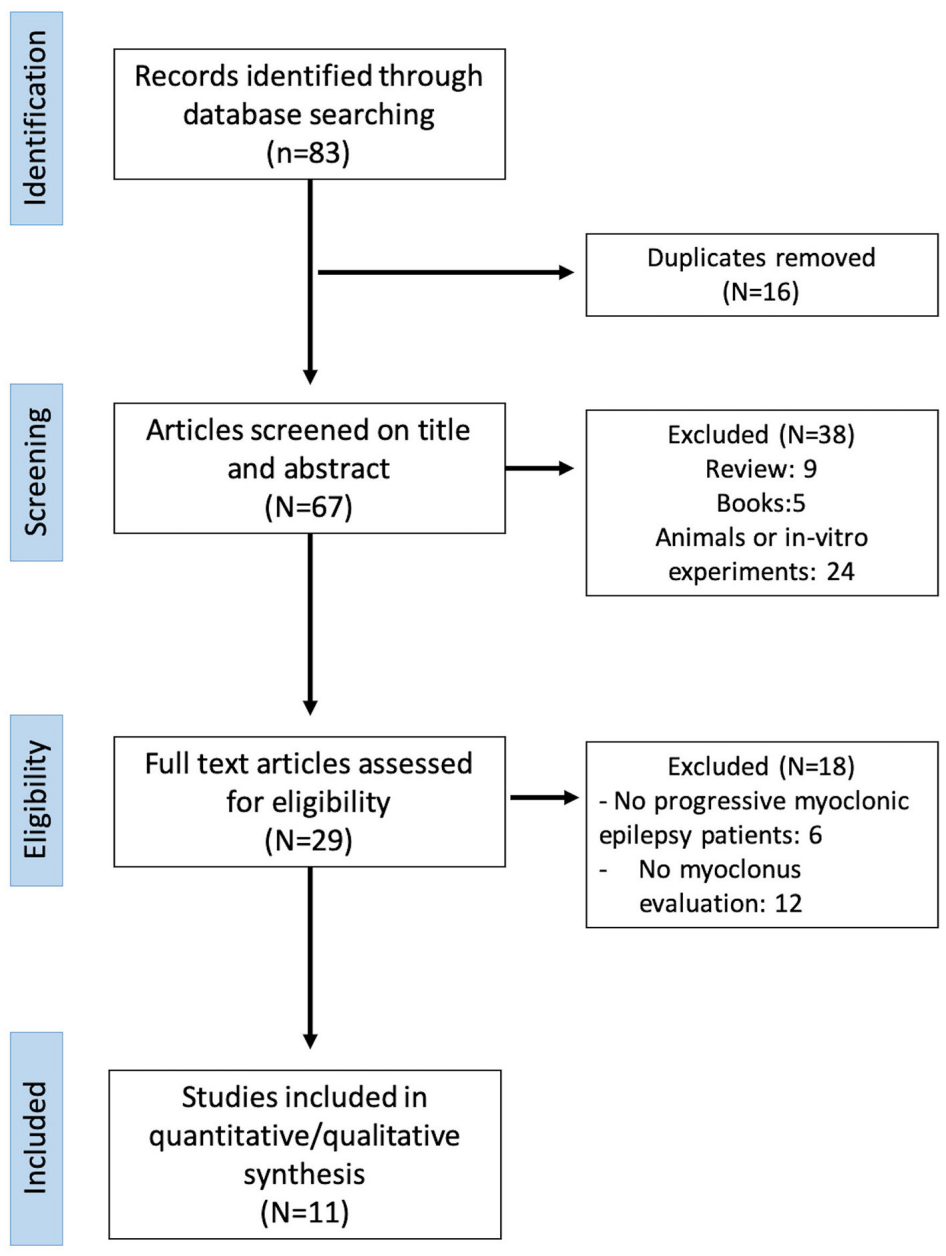
Preferred Reporting Items for Systematic Reviews and Meta-analyses (PRISMA) diagram of the systematic revision of manuscripts.

A systematic review was conducted by applying the Preferred Reporting Items for Systematic Reviews and Meta-analyses (PRISMA) guidelines (6) (Figure 2). Full-text articles and conference proceedings were selected from a comprehensive search of PubMed, Medline, Scopus, and Google Scholar databases. Keywords and their synonyms were combined in each database as follows: (“perampanel”) AND (“progressive myoclonic epilepsy” OR “Unverricht–Lundborg” OR “Lafora” or “MERRF” OR “Sialidosis” OR “Kufs” OR “AMRF” OR “ceroid lipofuscinoses” OR “BAFME” OR “Gaucher” OR “dentato-rubro-pallido-luysian atrophy”). No filter was applied on the publication data of the articles, and all results of each database were included up to February 2020. After removal of duplicates, all articles were evaluated through a screening of title and abstract by three independent reviewers (GA, MT, LR). The same three reviewers performed an accurate reading of all full-text articles assessing them for eligibility to this study, and they performed a collection of data to minimize the risk of bias. In case of disagreement among investigators regarding the inclusion and exclusion criteria, the senior investigator made the final decision.

We adopted the following inclusion criteria to include manuscripts in our review. The study must be an evaluation of the clinical impact of perampanel and have an abstract in English language. It must include clinical data to evaluate neurological changes induced by perampanel and must be published in a peer-reviewed journal. We also sorted available studies in the literature according to the following exclusion criteria. We excluded those studies including patients with myoclonic epilepsies other than PME and those not mentioning the perampanel effect on myoclonus. We also removed study designs that allowed concomitant antiseizure medication changes during the perampanel treatment trial and those studies conducted in animals or *in vitro* models. We also considered only original papers and excluded review manuscripts and books from our analysis.

### Data Extraction Process

Data extraction was executed on 83 articles (Figure 2). Data were extracted on the basis of the following checklist: authors, year, and type of publication (i.e., conference or full text), epilepsy syndrome. Sixteen articles were excluded because of duplication. Analysis of title and abstract caused the exclusion of 38 manuscripts not meeting inclusion and exclusion criteria (review, 9; books, 5; animals or *in vitro* experiments, 24). Further 18 manuscripts were excluded after reviewing the full papers (no progressive myoclonic epilepsy patients, 6; no myoclonus evaluation, 12). Eleven articles met the inclusion/exclusion criteria and were included in the review to provide the qualitative and quantitative analyses. Data were reported as follows (for details, see Table 2).

**Table 2:** Demographic and clinical data according to the diagnosis in the reviewed manuscripts.

**References**	**Pts *N***	**Diagnosis**	**Fem *N***	**Age Years, Mean ± sd (range)**	**Disease duration years Mean ± sd (range)**	**CoASMs Mean(range)**	**Perampanel mg/day Mean ± sd (range)**	**F-up *months***	**Drop out *N***	**Seizures pre-PER**	**Seizure responders *N***	**Myoclonus Improvement after-PER *N***	**ADL Improvement after-PER *N***	**Benefits until F-UP**
Canafoglia et al. (7)	18	ULD	7	39.6 ± 14.8	29.7 ± 12.8	3	4.7 ± 1.7	(4–6)	1	Yes	4/4	7/18	N/A	Yes
	12	Lafora disease	6	25.7 ± 10.8	11.2 ± 9.1	3	7.0 ± 3.0	(4–6)	1	Yes	11/11	1/12	N/A	Yes
	5	Sialidosis	4	40.0 ± 6.1	24.0 ± 7.4	3	4.4 ± 2.2	(4–6)	0	No	N/A	2/5	N/A	Yes
	1	KUFS	1	16	3	3	N.A.	(4–6)	1	Yes	1/1	1/1	N/A	Yes
	1	EPM7	0	19	7	3	12	(4–6)	0	No	N/A	0/1	N/A	Yes
	12	Undetermined	9	44.8 ± 17.5	24.2 ± 18.6	2	4.2 ± 1.6	(4–6)	2	Yes	2/2	7/12	N/A	Yes
Oi et al. (8)	7	ULD	4	44.3 ± 19.8(22–70)	N/A	4	2.9 ± 0.9	8.5 ± 16	0	N/A	N/A	7/7	7/7	Yes
	6	BAFME	4	60.5 ± 10.3(46–71)	N/A	(1–3)	1.6 ± 1.1 (0.5–3)	8.5 ± 16	0	N/A	N/A	2/5	5/5	Yes
	2	DRPLA	0	43 ± 4.2(40–46)	N/A	3	3	8.5 ± 16	0	N/A	N/A	2/2	2/2	Yes
	1	GD	0	34	N/A	2	3.5	8.5 ± 16	0	N/A	N/A	1/1	1/1	Yes
Crespel et al. (9)	12	ULD	6	(13–62)	27.6 ± 6.8 (5–52)	(1–6)	6	12.6 ± 7.6(3–21)	4	Yes	6/6	10/12	4/8	Yes
Assenza et al. (present)	5	ULD	0	40 ± 7.5	29.6 ± 7.0 (19–33)	(4–6)	6.4 ± 2.6 (4–10)	15 ± 2.6(12–19)	0	No	N/A	5/5	5/5	Yes
	3	Lafora	0	20 ± 4.5	10 ± 4.3 (7–15)	(3–4)	8 ± 2 (6–8)	15 ± 3 9(6–24)	0	Yes	1/3	1/3	1/3	No
	2	Sialidosis	2	22 ± 4	9.5 ± 4.9 (6–13)	2	4	4.5 ± 1.5(3–6)	0	Yes	1/2	2/2	2/2	Yes
	1	Undetermined	0	34	26	3	4	6	0	Yes	1/1	1/1	1/1	Yes
Goldsmith and Minassian (10)	10	Lafora	8	22.5	8.7 ± 7.3 (2–27)	(2–6)	6.7 (4–10)	10	3	Yes	4/8	5/7	0/7	Yes
Dirani et al. (11)	1	Lafora	1	15	3	0	10	7	0	Yes	1/1	1/1	1/1	Yes
Hu et al. (12)	1	Sialidosis	0	15	3	4	10	20	0	Yes	1/1	1/1	1/1	Yes
Oi et al. (13)	1	ULD	1	32	23	4	2	1	0	N/A	N/A	1/1	1/1	Yes
Schorlemmer et al. (14)	1	Lafora	1	21	7	7+ KD	10	4	0	Yes	1/1	1/1	1/1	Yes*
Shiraishi et al. (15)	1	DPRLA	0	13	N/A	4	0.8	3	0	Yes	1/1	1/1	1/1	Yes
Wong et al. (16)	1	CLP 2	1	3	N/A	6+KD	6	6	0	Yes	1/1	1/1	N.A.	Yes
Whole group	104	mixed	59			(2–8)			12		34/40	60/100	33/46	Yes

*N, number; Pts, patients; Fem, females; Sd, standard deviation; CoASMs, number of concomitant anti-seizure medications; PER, perampanel; ADL, activity daily living; F-UP, follow-up; KD, ketogenic diet; N./A., not applicable; ULD, Unverricht-Lundborg disease; CLP, ceroidolipofuscinosis; BAFME, benign adult familial myoclonus epilepsy; DPRLA, dentato-pallido-rubro-luisiana; GD, Gaucher disease; LAS, Lance-Adams syndrome*.

* *follow-up reported only for seizures*.

## Results

No randomized or controlled trials were found. All selected manuscripts were longitudinal retrospective case–control series. These involved 104 patients (59 female), of which 43 were affected by ULD, 27 by Lafora disease, eight by sialidosis, six by benign adult familial myoclonic epilepsy (BAMFE), one by Kufs, three by dentato-rubro-pallido-luysian atrophy (DPRLA), one by EPM7, one by Gaucher disease (GD), one by ceroid lipofuscinoses (CLP), and 13 by an undetermined PME. The included individuals were under treatment with zero to six concomitant ASMs before initiating perampanel. Twelve dropped out (11%). Follow-up ranged from 1 month to about 2 years. Clinical effects were evaluated after perampanel assumption at a dosage ranging from 0.8 to 12 mg/day. Manuscripts report was ordered by sample size.

Canafoglia et al. (7) described an Italian multicenter study on a heterogeneous sample of 49 patients with different forms of PMEs. The authors reported a myoclonus severity score for all patients, before perampanel treatment and after 4–6 months of continuous perampanel treatment. Patients with ULD or ULD-like phenotype was more likely to demonstrate improvement by perampanel treatment (*p* = 0.011) than patients with other PME diagnoses. Seventeen out of 49 patients presented with at least monthly seizures, and all of them reported a reduction in seizure frequency by >50%. Four patients dropped out for inefficacy/adverse events, while side effects occurred in 22 out of 49 (44.8%) patients, the most common one being irritability and drowsiness. Authors did not report outcomes on ADL.

Oi et al. (8) reported 16 cases of Japanese patients with PMEs with a follow-up of several months. In the manuscript, they also included two patients with drug-resistant myoclonus and the diagnosis of Lance-Adams syndrome, which was not included in our paper because it is not a progressive disorder. They described a clinical amelioration of both the myoclonus and ADL scores in 7/7 ULD, 2/5 benign adult familial myoclonus epilepsy (BAFME), 2/2 dentatorubral–pallidoluysian atrophy (DRPLA) patients, and in the only patient with Gaucher Disease. No data on changes in seizure frequency were provided.

Crespel et al. (9) explored the effect of perampanel on 12 ULD patients with a follow-up of 3–21 months. They homogeneously titrated perampanel dosage up to 6 mg/day in all patients. The six patients who still experienced bilateral tonic–clonic or myoclonic seizures stopped having seizures. Myoclonus had a significant improvement in 10/12 patients and ADL improved in 4/8. No dropouts were reported. Myoclonus improvement plateaued after a few months in two patients referred. However, they still retained a significant clinical effect at the last follow-up.

Goldsmith and Minassian (10) collected 10 young adults with Lafora disease taking perampanel 4–10 mg/day. Three patients withdrew due to inefficacy or side effects, while the remaining patients showed an improvement of seizures (4/6) and myoclonus (6/8 at 3 months follow-up, 4/8 at 10 months follow-up) but not ADL.

Other two patients with Lafora disease were reported in the manuscripts of Dirani (11) and in that of Schorlemmer (14). Both of them ameliorated in myoclonus and ADL. Shorlemmer further reported a seizure improvement with achieving seizure freedom.

Hu and colleagues (12) described an adolescent with sialidosis who presented a drastic stop of myoclonus (from more than 100 episodes per day), a complete seizure-free status, and an improvement of ADL lasting until 20 months follow-up.

Shiraishi et al. (15) reported an adolescent with DRPLA with strong clinical effect, wiping out seizures and myoclonus and ameliorating ADL.

Wong et al. (16) reported a child with CLP type 2, treated with six ASMs and ketogenic diet and had a significant reduction in seizures and myoclonus under perampanel treatment.

### Myoclonus

**Figure 3:**
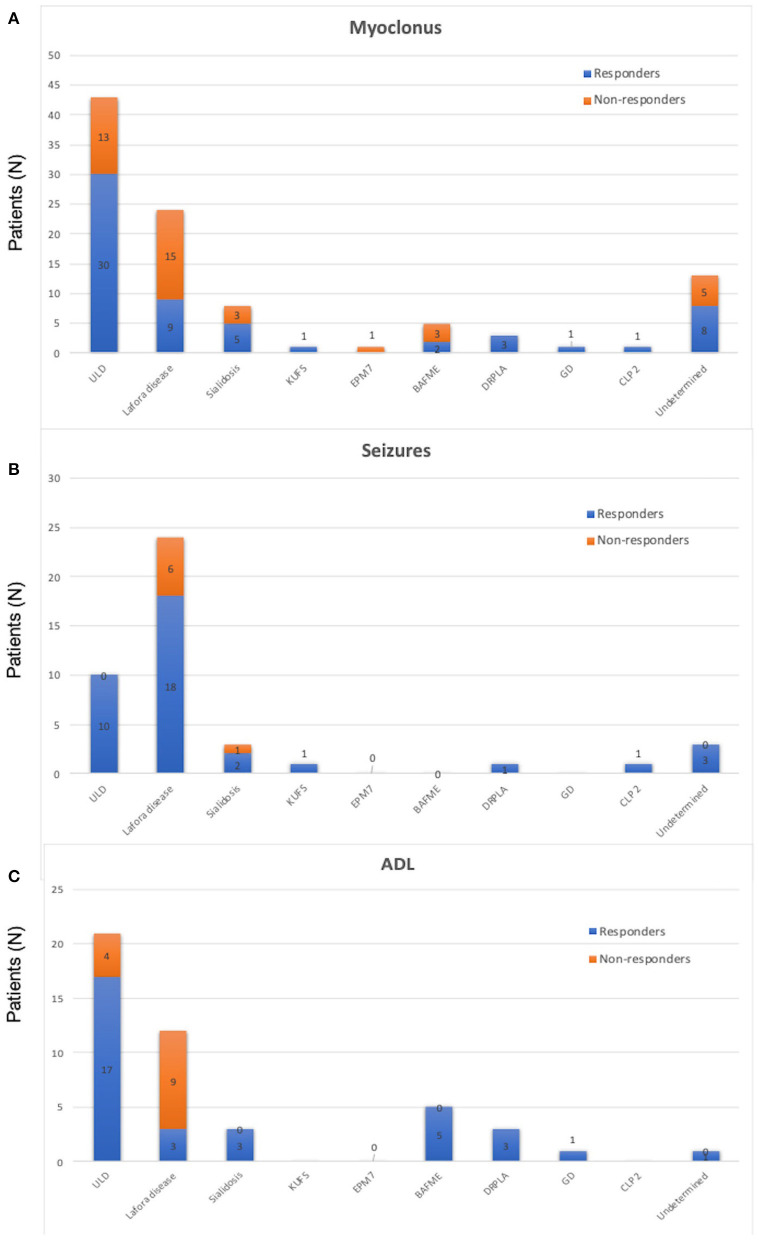
**(A)** Frequency of people experiencing a significative myoclonus reduction in the whole sample of the reviewed 104 cases of progressive myoclonic epilepsies. Resp, responders; N-Resp, non-responders; ULD, Unverricht–Lundborg disease; EPM7, epilepsy progressive myoclonic 7; BAFME, benign adult familial myoclonus epilepsy; DRPLA, dentatorubral pallidolusyan atrophy; CLP 2, ceroid lipofuscinosis type 2. **(B)** Frequency of people experiencing a significative seizure reduction in the whole sample of the reviewed 104 cases of progressive myoclonic epilepsies. Resp, responders; N-Resp, non-responders. ULD, Unverricht–Lundborg disease; EPM7, epilepsy progressive myoclonic 7; BAFME, benign adult familial myoclonus epilepsy; DRPLA, dentatorubral pallidolusyan atrophy; CLP 2, ceroid lipofuscinosis type 2. **(C)** Frequency of people experiencing a significative amelioration in activities of daily living activities in the whole sample of the reviewed 104 cases of progressive myoclonic epilepsies. Resp, responders; N-Resp, non-responders; ULD, Unverricht–Lundborg disease; EPM7, epilepsy progressive myoclonic 7; BAFME, benign adult familial myoclonus epilepsy; DRPLA, dentatorubral pallidolusyan atrophy; CLP 2, ceroid lipofuscinosis type 2.

Quantitative/qualitative analysis of myoclonus was available in 100 PME patients. Sixty of them obtained an action myoclonus improvement (60%). When considering patients according to their diagnosis, we found a myoclonus amelioration in 70% of ULD, 38% of Lafora, 63% of sialidosis, 100% of KUFS, 0% of EPM7, 40% of BAFME, 100% of DPRLA, 100% of GD, 100% of CLP 2, and 62% of undetermined patients (frequencies in Figure 3A).

### Seizures

Thirty-six out of the 43 patients (92%) with available seizure frequency and at least one seizure in the last trimester before perampanel introduction had a significant reduction in seizures frequency (a seizure reduction by >50% was defined as seizure responders; Figure 3B). All patients with ULD (10/10; 100%), 18/24 (75%) patients with Lafora disease, 2/3 sialidosis (66.6%), 1/1 of KUFS (100%), 3/3 undetermined (100%), 1/1 DRPLA (100%), and 1/1 CLP 2 (100%) were seizure responders.

### ADL

Thirty-three out of 46 patients (72%) with available scores reported an improvement of ADL after perampanel introduction. In particular, 12/16 ULD (75%), 5/5 (100%) BAFME, 3/3 (100%) DPRLA, 1/1 (100%) GD, 3/12 (25%) Lafora disease, 3/3 (100%) sialidosis, and 1/1 (100%) undetermined patients were described as ameliorated in ADL by their physicians (Figure 3C).

## Discussion

In the present manuscript, we reported our real-life experience on the effectiveness of perampanel on myoclonus, ADL, and seizures in an Italian multicenter case series of 11 individuals with diverse PMEs (ULD, Lafora, sialidosis, and undetermined). We recorded a significant amelioration of all these clinical features in the majority of our patients; thus, we decided to add our cases to an original systematic review of the literature on the same topic. We did not find any prospective clinical trial but only retrospective case series, most of them reporting a small number (<20) of patients and often only one patient. Thus, the evidence of the presented results is limited, and prospective trials are to be encouraged. Furthermore, action myoclonus and disability on ADL were not always assessed by validated clinical scales, so that we reported only the qualitative amelioration that could be inferred by each manuscript. However, given the rarity of PMEs, we think that the systematic review of all available data could provide useful suggestions for clinical practice.

Actually, in a group of more than 100 people with different PMEs, we found that, besides the well-known role of perampanel in controlling generalized seizures (4, 17), more than half of the patients received an amelioration of action myoclonus and, consequently, of their disability in ADL. These results were mainly obtained because the myoclonus amelioration allowed the patient to move from the wheelchair to a standing position or to perform some steps without falls.

Disease Group Effect on Myoclonus, Disability, and Seizures.

We aimed to understand whether specific diagnoses of PME may account for a different clinical response to perampanel therapy. Among 10 different PME disorders, we found a mean response of myoclonus and disability in about 60% of patients. The proportion of clinical amelioration seems constant through the different diagnoses, even if most of them had a small numerosity, which does not allow to draw a reliable correlation between genotype and drug response. However, the group of ULD including 43 patients demonstrated an action myoclonus improvement in 70% of patients, while the Lafora disease group including 24 patients reported a myoclonus improvement in 38% of them.

ULDs were described as ameliorated in their independence in ADL in 81% of cases while Lafora patients in only 25% of patients. Beneficial results were also reported in the smaller groups with different PMEs.

Furthermore, the antiseizure effect of perampanel in PME showed a similar trend. ULD patients were those receiving the strongest benefit (all the residual GTCSs were wiped out), while 75% of persons with Lafora disease presented a significant GTCS frequency reduction (>50%). A very good GTCS response was also recorded in the smaller groups.

The differences recorded between ULD and Lafora groups with regard to all parameters (myoclonus, seizure, and disability response) could be explained not only by the different group numerosity but also by the different clinical phenotype, which is more severe in the Lafora disease (if we leave ULD patients with compound heterozygous mutations out, usually presenting more invalidating clinical deficits) (18, 19).

Most of our PME patients (8 out of 11) retained the clinical benefit until the last follow-up, which was clinically significant, suggesting that the perampanel effect on myoclonus could be produced by a specific pathogenetic antimyoclonic effect rather than by the mere antiseizure effect, which is known to commonly undergo honeymoon effect in drug-resistant epileptic patients (20).

### Perampanel Discontinuation

In our revised population, the proportion of patients withdrawing perampanel was similar to that reported in the randomized trial assessing the clinical efficacy and safety of perampanel in primary generalized tonic–clonic seizures in genetic generalized epilepsy. However, none of our patients dropped out for intolerable side effects, which were the only reason of dropout in the treated arm of the randomized trial (about 16% of patients had dizziness and fatigue). PME patients exclusively withdrew perampanel because of inefficacy in about 11% of cases. Our population was heavily medicated by antimyoclonic treatments (antiseizure drugs and ketogenic diet), which were mixed up to eight treatments in the same patient. The overmedication and the cognitive impairment might account for the lack of reports of significant side effects compromising the patients' compliance with the perampanel adjunctive therapy. However, it is worth noting that many of the reviewed PME patients received low doses of perampanel, confirming that perampanel efficacy is already manifested at low doses, however is not dose dependent. This pharmacokinetic feature of perampanel became evident only after several years after commercialization, and, at present, the protocol of the reported randomized trial suggested that the patients should receive a titration up to 8 mg/day. Furthermore, the initial indication was to use a very fast titration (up to 8 mg/day in 4 weeks), while the pharmacodynamic of perampanel (half-life of approximately 105 h) might suggest very slow increase in dosage. These considerations might explain the different reasons of withdrawal of PME patients with respect to those of the randomized trial.

### Pathophysiology of Myoclonus and Perampanel Implications

Different forms of PME can present subtle differences in etiology and, thus, neurophysiology of their cardinal clinical sign, i.e., the myoclonus. However, all PMEs present different grades of multifocal reflex (action-induced) myoclonus. This type of myoclonus is assumed to be cortically generated, since it is typically associated with “subtle” central electroencephalogram (EEG) changes that can be studied using EEG–electromyography relationship analysis (21), but it is likely generated by a global hyperexcitability of the whole cortico-subcortical sensory–motor network (22). Moreover, cortical myoclonus is coupled with neurophysiological features reflecting neocortical hyperexcitability, such as “giant” evoked potentials and enhanced long-loop reflexes (23). In this scenario, a significant experiment was provided by Oi and colleagues (8), the authors of one of the manuscripts included in our review. They included in their work a neurophysiological study of the effects of perampanel on sensorimotor circuits by means of scalp somatosensory-evoked potentials (SSEPs). SSEPs are able to reliably estimate cortical excitability in epileptic patients (24, 25). They found that perampanel induced a decrease in P25 and N33 potentials, which are usually the SSEPPs components reported as “giant” in PME and other myoclonic epilepsies (26, 27). Actually, P25 and N33 potentials represent the intracortical signal amplification occurring by the propagation from primary sensitive to primary motor areas. According to these authors, the polysynaptic origin of P25 and N33 would explain why perampanel mainly acts only on these waves and not on the N20, which is generated by the monosynaptic thalamocortical afferents to the primary sensory cortex. From a biological point of view, cortical myoclonus is also favored by a deficit in the GABAergic tone (28) and thus by an unbalance of excitatory and inhibitory synapses favoring the former. Oi's findings might be explained by the specific pharmacodynamic of perampanel. Perampanel is a selective inhibitor of postsynaptic AMPA receptors; thus, the mechanism of action is purely synaptic and specific on glutamatergic excitatory neurons, and it is different from other unspecific sodium-channel blockers used as ASM, which could also impair non-synaptic action potential propagation. These features are likely responsible for the antiseizure effect of perampanel as demonstrated by its efficacy in drug-resistant focal epilepsies and also for inhibiting cortico-subcortical neural circuits of signal amplification and topographical diffusion (4, 29). This also likely yields to perampanel efficacy in primary generalized tonic–clonic seizures and in myoclonic disorders.

### Limitations

We are aware of the relevant limitations of our review. First, the scientific evidence remains limited, as it did not include any prospective or controlled study. Furthermore, the lack of uniformly adopted specific scores for myoclonus and ADL assessment did not allow to produce any quantification of the perampanel effects. Actually, while UMRS is the gold standard scale to assess the impact of myoclonus in neurological patients, it is quite long and challenging for the examiner and the patient. It is a detailed scale that consists of a patient questionnaire and a score sheet with 72 items for the clinicians to be completed after reviewing a videotape of the patient while doing several neurological tests. In this light, the simplified myoclonus rating scale (SMRS) allows a more easy and practical use, particularly for testing patients during an outpatient examination, even if it only quantifies the interference of myoclonus over activities of daily life.

Furthermore, in several manuscripts, the action myoclonus and disability assessment were provided only by means of the qualitative subjective report of the physician.

The low probability of publication of negative results about the treatment with perampanel in PME is another possible bias overestimating our positive results.

## Conclusions

We reported an original case series and a systematic review of the perampanel effectiveness on myoclonus and disability in patients affected by the rare forms of progressive myoclonic epilepsies. The qualitative analysis of our results showed, although with a limited scientific evidence, that perampanel could be an adequate treatment for these patients because most of them, independently from the specific form of the disorder, reported a significant improvement of action myoclonus and consequent independence.

In conclusion, perampanel represents a valid antiseizure and symptomatic therapy, widening the restricted armamentarium available for progressive myoclonic epilepsies, where some commonly used ASMs (Na blockers) are not recommended.

## Data Availability Statement

The original contributions generated for this study are included in the article/Supplementary Material, further inquiries can be directed to the corresponding author/s.

## Author Contributions

GA: original case descriptions, systematic revision, and manuscript drawing and coordination. CN, GD, AD'A, AV, and AM: original case descriptions. MT and LR: systematic revision. JL: figures and video. VD and LB: senior supervisor. AC: original case description, critical revision, and manuscript coordination. All authors contributed to the article and approved the submitted version.

## Conflict of Interest

The authors declare that the research was conducted in the absence of any commercial or financial relationships that could be construed as a potential conflict of interest.

## Supplementary Material

Supplementary Video 1 The video shows the action myoclonus response in a patient with sialidosis type 1 after treatment with perampanel (patient 6, Table 1).
